# Distinct immune stimulatory effects of anti-human VISTA antibodies are determined by Fc-receptor interaction

**DOI:** 10.3389/fimmu.2022.862757

**Published:** 2022-07-28

**Authors:** Sven Mostböck, Helen Haixia Wu, Timothy Fenn, Bettina Riegler, Susanne Strahlhofer, Yining Huang, Gale Hansen, Rachel Kroe-Barrett, Iñigo Tirapu, Anne B. Vogt

**Affiliations:** ^1^ Cancer Immunology and Immune Modulation, Boehringer Ingelheim RCV GmbH & Co KG, Vienna, Austria; ^2^ Biotherapeutics Discovery, Boehringer Ingelheim Pharmaceuticals, Inc., Ridgefield, CT, United States

**Keywords:** VISTA, antibody, Fc-receptor, immunotherapy, immune checkpoint

## Abstract

VISTA (PD-1H) is an immune regulatory molecule considered part of the next wave of immuno-oncology targets. VISTA is an immunoglobulin (Ig) superfamily cell surface molecule mainly expressed on myeloid cells, and to some extent on NK cells and T cells. In previous preclinical studies, some VISTA-targeting antibodies provided immune inhibitory signals, while other antibodies triggered immune stimulatory signals. Importantly, for therapeutic antibodies, the isotype backbone can have a strong impact on antibody function. To elucidate the mode of action of immune stimulatory anti-VISTA antibodies, we studied three different anti-human VISTA antibody clones, each on three different IgG isotypes currently used for therapeutic antibodies: unaltered IgG1 (IgG1-WT), IgG1-KO (IgG1-LL234,235AA-variant with reduced Fc-effector function), and IgG4-Pro (IgG4- S228P-variant with stabilized hinge region). Antibody functionality was analysed in mixed leukocyte reaction (MLR) of human peripheral blood mononuclear cells (PBMCs), as a model system for ongoing immune reactions, on unstimulated human PBMCs, as a model system for a resting immune system, and also on acute myeloid leukemia (AML) patient samples to evaluate anti-VISTA antibody effects on primary tumor material. The functions of three anti-human VISTA antibodies were determined by their IgG isotype backbones. An MLR of healthy donor PBMCs was effectively augmented by anti-VISTA-IgG4-Pro and anti-VISTA-IgG1-WT antibodies, as indicated by increased levels of cytokines, T cell activation markers and T cell proliferation. However, in a culture of unstimulated PBMCs of single healthy donors, only anti-VISTA-IgG1-WT antibodies increased the activation marker HLA-DR on resting myeloid cells, and chemokine levels. Interestingly, interactions with different Fc-receptors were required for these effects, namely CD64 for augmentation of MLR, and CD16 for activation of resting myeloid cells. Furthermore, anti-VISTA-IgG1-KO antibodies had nearly no impact in any model system. Similarly, in AML patient samples, anti-VISTA-antibody on IgG4-Pro backbone, but not on IgG1-KO backbone, increased interactions, as a novel readout of activity, between immune cells and CD34+ AML cancer cells. In conclusion, the immune stimulatory effects of antagonistic anti-VISTA antibodies are defined by the antibody isotype and interaction with different Fc-gamma-receptors, highlighting the importance of understanding these interactions when designing immune stimulatory antibody therapeutics for immuno-oncology applications.

## Introduction

VISTA (V-domain Ig–containing suppressor of T cell activation, also called PD-1H) is a surface-expressed protein with multiple roles. It has mainly been studied for its functions in immune regulation and as a new target for immune-modulatory antibodies, as recently reviewed (1). VISTA acts as both receptor and ligand. Its relevant functional binding partner has not been fully clarified yet; possibly VISTA can interact with different partners under respective suitable circumstances. PSGL-1 has been described as a binding partner at low-pH conditions (2), which might for example occur in distinct compartments of activated lymph nodes (3). VSIG-3, another putative functional partner (4, 5), seems to bind best at physiological pH (6). Furthermore, VISTA was observed to form homophilic interactions, to be part of the BMP4-receptor, and to interact with VSIG8, Galectin-9 and Syndecan-2 (7–11). Which of these binding partners is the most relevant for immune modulation in humans remains to be understood.

VISTA functions as a suppressor of T cell responses as both receptor and ligand. *In vitro* studies and *in vivo* mouse models point to VISTA as a major checkpoint molecule preventing activation of naïve T cells. Several presumably agonistic anti-VISTA antibodies, and also recombinant VISTA protein (supporting VISTA’s dual role as receptor and ligand), have been shown to suppress activation of naïve T cells and to prevent auto-immune reactions (12–16), while some presumably antagonistic antibodies led to increased levels of auto-immune and allergic diseases in mouse models (16–18). Thus, VISTA has been discussed as being distinct from most other checkpoint inhibitors as it keeps naïve T cells inactive, instead of suppressing T cells at later stages after their activation (12).

There are conflicting studies about VISTA’s function in regulating myeloid cells. VISTA overexpression on human monocytes led to increased levels of spontaneous cytokine production as well as phagocytosis and furthermore to a stronger antigen-based T cell activation, while VISTA-knock down on monocytes reduced subsequent T cell activation (19) – an unexpected finding given that VISTA is generally observed to be an immune suppressive ligand for T cells. Similarly, VISTA-deficiency reduced inflammation in mouse models for collagen antibody-induced arthritis and for immune complex-mediated glomerulonephritis (20, 21). Others, however, observed suppression of myeloid cell function by VISTA: VISTA-deficient murine myeloid cells responded faster and with stronger cytokine release to activation, and antagonistic anti-VISTA-antibody increased macrophage function (9, 22–25). Similarly, an agonistic VISTA antibody was shown to shift human monocytes towards an anti-inflammatory state *in vitro* (26).

VISTA is considered part of a next wave for immunotherapy in oncology. Mouse studies demonstrated that antagonistic anti-VISTA antibodies reduced tumor burden and/or increased survival (2, 6, 27–34). Additionally, some human cancers, such as NSCLC, CRC and AML, demonstrate a high level of VISTA expression, mostly on myeloid cells, but also on T cells and tumor cells (1). Interestingly, VISTA was found to be upregulated in tumor tissue following standard immunotherapy of metastatic melanoma patients (35) and prostate cancer patients (36), suggesting VISTA as a possible combination partner for standard therapies.

In this study, we present findings on the impact of the isotype of the antibody backbone on the functional effects of immune stimulatory anti-human VISTA antibodies. In human *in vitro* systems, these antibodies led to increased cell activation and cytokine release by T cells and myeloid cells. The antibody activity on the specific cell type was determined by the antibody backbone. While unaltered IgG1 (IgG1-WT) backbone led to activation of both, T cells and myeloid cells, an IgG4-Pro (stabilized Ser228Pro variant) backbone stimulated only the function of T cells. Furthermore, the effects of the anti-VISTA-antibodies were shown to depend on interaction with particular Fc-receptors. These findings highlight the importance of understanding antibody isotype effects and the role of Fc-receptor interaction on their mode of action when designing immune stimulatory antibody therapeutics for therapeutic applications.

## Materials and methods

### Generation and biophysical characterization of a novel anti-human VISTA antibody

Wild type or AlivaMab mice (under license of AlivaMab Discovery Services) were immunized in house with mammalian-expressed human recombinant VISTA extracellular domain fused with human Fc1 protein. Serology was assessed by ELISA with recombinant human VISTA-His or cynomolgus VISTA-His protein (R&D Systems). Splenocytes from selected mice were harvested and screened for VISTA-binding. Antibody sequences with cross-reactivity to cynomolgus VISTA were produced in Chinese hamster ovarian (CHO) cells *via* high through-put (HTP) expression. Clones were confirmed by assessing binding to human VISTA-expressing THP-1 cells by flow cytometry, and converted to chimeric human IgG4-Pro backbone with a Ser228Pro change alteration in order to prevent Fab-arm exchange (37). These IgG4-Pro monoclonal antibodies were expressed in CHO cells transiently and then purified following standard methods with modifications (38, 39). These IgG4-Pro monoclonal antibodies were profiled in functional assays (described below) and biophysical characterization assays, such as hydrogen-deuterium exchanged mass spectrometry analysis (HDX) for epitope analysis and non-specific binding (NSB) assay, as described previously (40). Based on experimental data and sequence liability analysis, two example clones, J014 and E008, were selected for this study. Clone VSTB112 has been published before (6, 41).

These three VISTA antibody clones and isotype control antibody (anti-TNP) were formatted onto three different human IgG backbones: wild-type IgG1 (IgG1-WT), IgG4-Pro, and IgG1 with LALA (LL234,235AA) alterations that practically abolish Fc-receptor binding (IgG1-KO) (42). These chimeric antibodies were expressed in CHO cells and purified using standard methods with modifications, as described before (40).

### Mixed leukocyte reaction (MLR)

Cryo-preserved human PBMCs (Stemcell Technologies) were thawed and donor pairs (250,000 cells each from two different donors) added per well in round-bottom 96-well plates. Test antibodies were added to a final concentration of 10 µg/ml. Cells were cultured in 200 µl CellGro DC Medium (CellGenix) per well in an incubator at 37°C with 5% CO2. Cells and supernatant were harvested on day 4 or 5 of culture. Cells were analysed by flow cytometry; cytokine levels were assayed using commercial kits from Meso Scale Discovery.

### Monocyte activation assay

Cryo-preserved human PBMCs (Stemcell Technologies) were thawed and 200,000 cells from one donor added per well in round-bottom 96-well plates. Test antibodies were added to a final concentration of 1 µg/ml. Cells were cultured in 200 µl RPMI-1640 (supplemented with 5% GemCell human serum AB/Gemini, 1 x non-essential amino acids/Gibco-Invitrogen, 1 x sodium pyruvate/Gibco-Invitrogen, 10 mM HEPES/Affymetrix-Invitrogen, 1 x Penicilin-Streptomycin/Gibco-Invitrogen) per well in an incubator at 37°C with 5% CO2. Cells and supernatant were harvested after overnight culture. Cells were analysed by flow cytometry; cytokine levels were assayed using commercial kits from Meso Scale Discovery. For some experiments, cell populations were enriched from PBMCs by magnetic bead separation with negative selection following manufacturer’s instructions (Stemcell Technologies).

### Fc-receptor blockade

For Fc-receptor blockade, cells were added to the wells in a volume of 100 µl medium. Then, Fc-receptor-blocking reagents (depending on experiment: LEAF anti-human CD16 antibody, clone 3G8, Biolegend; LEAF anti-human CD64 antibody, clone 10.1, Biolegend; functional grade anti-human CD32 antibody, clone 6C4, eBiosciences/Invitrogen; InVivoMAb anti-human CD32a antibody, clone IV.3, BioXCell; NA/LE human BD Fc Block, BD Biosciences; pure anti-human CD16 antibody, clone REA423, Miltenyi Biotec) or respective isotype controls were added to a concentration of 25 µg/ml in 150 µl volume per well, and plates were incubated on wet ice for 30 min. Then, anti-VISTA antibodies were added and cells cultured as described above. Blocking reagents remained in the culture for the whole culture duration.

### Flow cytometry

Cells were washed in FACS Buffer (PBS + 2% FCS + 0.01% sodium acetate) for monocyte activation assay, or PBS for MLR, and Fc-receptor blocking agent (human TruStain FcX, Biolegend) was added. Following 10 min incubation at 4°C, fluorochrome-labeled antibodies against cell surface molecules (eBioscience/Invitrogen, Miltenyi Biotec, Biolegend, BD Biosciences, United States Biological) were added and incubated for 20-30 min at room temperature or at 4°C. For analysis of MLR cultures, cell surface staining included a live/dead cell discriminator (fixable viability dye, Biolegend). After incubation, cells were washed with FACS Buffer and resuspended in FACS Buffer or fixation reagent (FluoroFix, Biolegend).

For analysis of Ki67-expression, FACS Buffer did not contain sodium acetate. Following staining of the surface antigens, cells were washed in FACS Buffer and resuspended in Buffer A of the Human FoxP3 Buffer Set (BD Biosciences). Tubes were shortly vortexed, incubated at room temperature for 10 min, washed in FACS Buffer, and cells resuspended in Buffer C. Tubes were shortly vortexed, incubated at room temperature for 30 min, and washed in FACS Buffer. Human TruStain FcX was added and tubes incubated for 5 min at room temperature. Fluorochrome-labeled anti-Ki67-antibody (Miltenyi Biotec) was added and tubes incubated for 30 min at room temperature, after which tubes were washed with FACS Buffer.

### Pharmacoscopy analysis of human AML samples

Leftover bone marrow (BM) or peripheral blood (PB) AML samples were collected after routine diagnostics under approved ethical protocols (EK:1447/2017) and informed patient consent. Mononuclear cells were purified by lymphoprep (Axis Shield) density gradient following manufacturer directions. Cells were used fresh, or were frozen into aliquots in FBS containing 10% DMSO and stored at 80°C. Frozen samples were rapidly thawed at 37°C and washed once in media containing FBS, and incubated with 45 units/ml DNase for 30 minutes at room temperature prior to another washing step in media. Experiments with anti-VISTA antibodies were conducted in CellGenix GMP DC media (CellGenix GmbH), supplemented with 10% FBS and 0.1% penicillin/streptomycin. Cells were plated at approximately 20,000 per well (400,000 cells/ml) in 384-well imaging plates (PerkinElmer, Cell Carrier Ultra), and incubated with 10 µg/ml anti-VISTA antibody or controls for several days, as documented in the figure legend. Plate fixation, imaging, and analysis was performed as described previously (43, 44). AML cancer cells were identified with anti-CD34, anti-CD117, effector cells with anti-CD3 (BD Biosciences) conjugated with GFP, phycoerythrin (PE), or allophycocyanin (APC).

### Statistical analysis

Data graphs and statistical analyses were generated with GraphPad Prism 9.3.1 (GraphPad Software, LLC). A paired ratio t-test was used for two group analysis. For ratio-analysis of multiple groups, data were log-transformed and analysed using a paired one-way ANOVA with Dunnett’s (anti-VISTA-antibodies compared to respective isotype) or Tukey’s multiple comparison test, or a paired two-way ANOVA using Sidak’s multiple comparison test. For **Figure 4B**, not all cytokine read-outs were performed for all subjects; thus, a two-way ANOVA was not possible and data were analysed by a paired mixed-effects model with Sidak’s multiple comparisons test. For **Figures 6A, C**, the log2 interaction scores from each experiment were fit to a Poisson regression generalized linear model (GLM, calculated with package stats in R version 3.6.1) using the sample, time, and simulation as variables, in order to determine the impact of J014-IgG4-Pro or J014-IgG1-KO versus isotype response. For **Figure 6B**, statistics were calculated package ggpubr version 0.3.0 in R 3.6.1. * in graphs indicates p<0.05.

## Results

### Generation of anti-human VISTA antibodies

From the serology analysis, about 290 positive binding clones with cynomolgus cross-reactivity were identified. Approximately 40 clones were confirmed as positive binders in the flow cytometry binding assay to human VISTA-expressing THP-1 cells. Based on functional screening in MLR and general biochemical assessment, two representative clones, J014 and E008, as well as the published clone VSTB112 (6, 41) were selected for further studying the nature of VISTA antibody pharmacology. The biophysical characterization data for the three clones on IgG4-Pro backbone demonstrates different affinities, yet a similar coverage (amino acids 22-36 and 115-134) of the extracellular domain (ECD) of human VISTA (**Table 1**).

**Table 1:** The biophysical characterization of anti-VISTA-antibodies on IgG4-Pro backbone.

Clone	J014	E008	VSTB112
K_D_ to human VISTA (nM)	>100	1.1	0.90
K_D_ to cynomologus VISTA (nM)	44	1.0	1.1
K_D_ to mouse VISTA (nM)	No binding at 100 nM		
Non-specific binding	No binding to either positively or negatively charged surfaces		
HDX protection	main: 22-36,	main: 22-36,	main: 22-36,
(VISTA ECD positions)	115-134	115-134	115-134,
	weak: 37-50	weak: 37-50	weak: 37-50

### VISTA antibody effects on ongoing immune responses depend on CD64

To study VISTA’s role in ongoing immune responses, we used mixed leukocyte reactions (MLR) of total human PBMCs isolated from two different donors, thereby allowing interaction of multiple immune cell types. In MLR, T cells react to non-matching allogeneic MHCs of the respective other donor, resembling the regular T cell receptor activation by specific MHC-peptide complexes. Only a few alloreactive T cells respond, and those interactions can be expected to vary in affinity and interaction strength, mirroring natural immune responses. We added the anti-VISTA antibodies directly at the start of the culture, thus modulating VISTA already at an early timeframe of the immune response.

**Figure 1:**
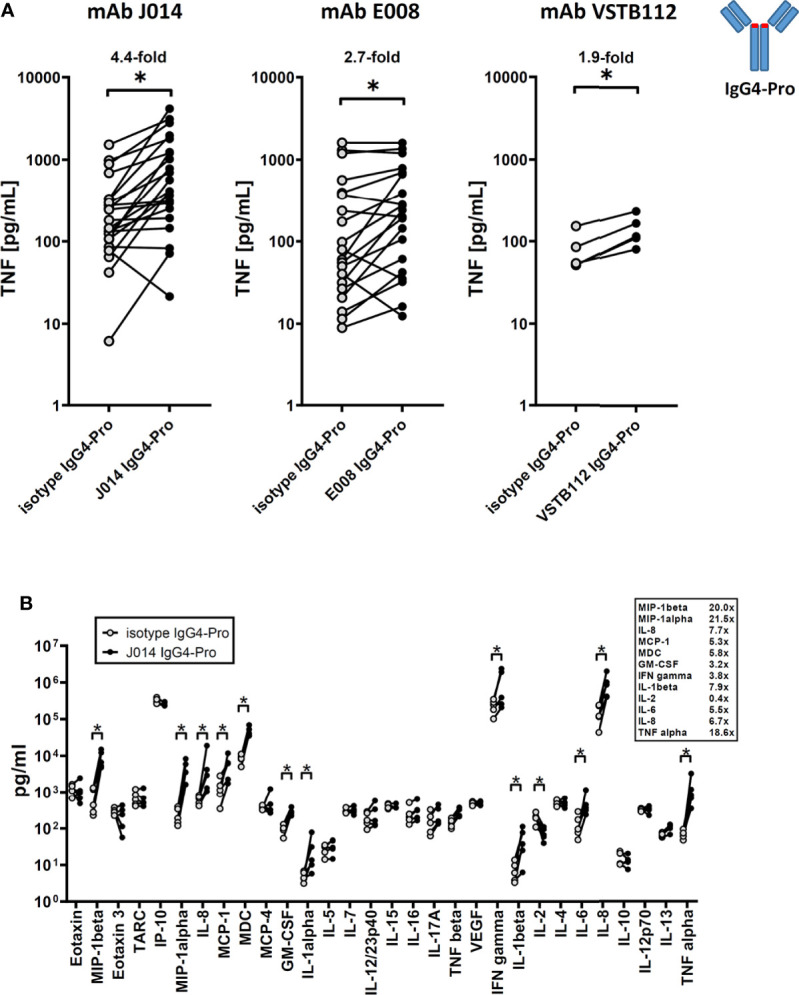
anti-VISTA antibodies enhance ongoing immune responses (MLR). PBMCs of donor pairs (two different donors) were co-cultured to induce a mixed leukocyte reaction (MLR) and treated with anti-VISTA-antibodies on IgG4-Pro backbone. **(A)** TNF levels in MLR culture supernatant treated with three different antibody clones (J014, E008, VSTB112; IgG4-Pro). Data is combined from multiple experiments, thus not showing the same number of data points for all antibody clones. Each connected symbol is a separate donor pair, some of which were used for testing multiple clones. Mean fold-changes between antibodies and respective isotype controls are indicated. **(B)** Panel of cytokines and chemokines in MLR cultures treated with anti-VISTA-antibody J014-IgG4-Pro. Data is from one experiment, each connected symbol represents a donor pair. * indicates p < 0.05.

**Figure 2:**
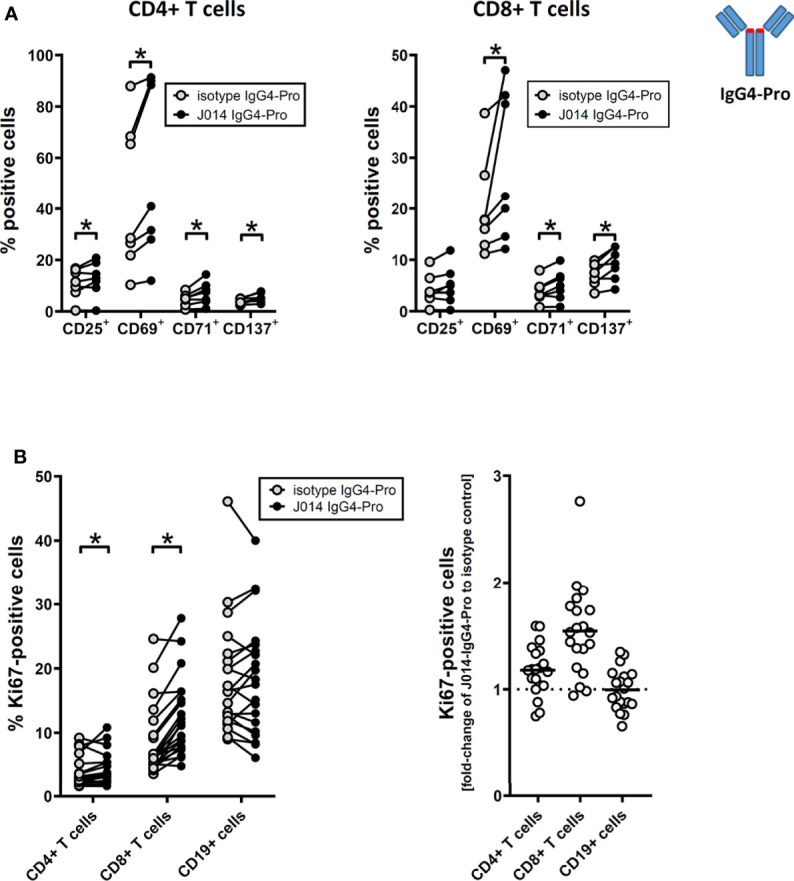
anti-VISTA antibodies enhance adaptive T cell response in MLR. PBMCs of donor pairs (two different donors) were co-cultured to induce a mixed leukocyte reaction (MLR) and treated with anti-VISTA-antibody J014 on IgG4-Pro backbone. **(A)** Impact of J014-IgG4-Pro on T cell activation markers of CD4+ T cells (left panel) and CD8+ T cells (right panel) in MLR cultures. Data is combined from multiple experiments; each connected symbol is a separate MLR donor pair, or a distinct donor of a donor pair. **(B)** Impact of J014-IgG4-Pro on the proliferation marker Ki67 in cells in MLR cultures. Ki67-levels are shown as percentage of positive cells in CD4+ T cells, CD8+ T cells and CD19+ B cells (left panel), and also as fold-change of the percentage of Ki67-positive cells comparing J014-IgG4-Pro-treatment with isotype control (right panel). Data is from one experiment, each symbol represents a donor pair. * indicates p < 0.05.

All three anti-VISTA antibody clones on IgG4-Pro, J014, E008 and VSTB112, led to increased levels of soluble TNF (**Figure 1A**; J014: mean 4.4-fold increase, E008: mean 2.7-fold increase, VSTB112: mean 1.9-fold increase). Using VISTA-antibody J014-IgG4-Pro as representative antibody, we observed that in addition to TNF, many other cytokines and chemokines, such as IFN-gamma, IL-5, IL-6, MIP-1-beta and IL-8, were increased (**Figure 1B**). Furthermore, the T cell activation markers CD25, CD69, CD71 and CD137 (**Figure 2A**), as well as the proliferation marker Ki67 (**Figure 2B**), were increased for CD4+ and, even more so, CD8+ T cells in cultures treated with VISTA antibody on IgG4-Pro backbone. In comparison, CD19+ cells did not show increased levels of proliferation (**Figure 2B**), demonstrating that the effects, at least on cell proliferation, were T cell specific.

**Figure 3:**
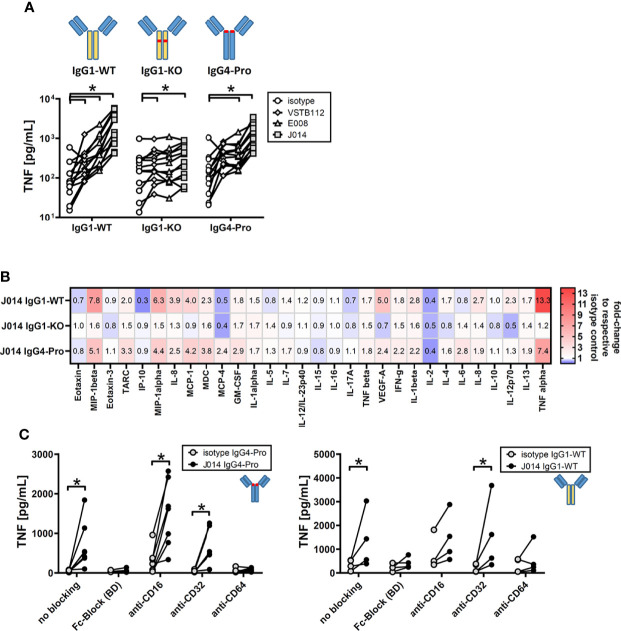
Fc-receptor dependency of anti-VISTA antibodies during MLR. PBMCs of donor pairs (two different donors) were co-cultured to induce a mixed leukocyte reaction (MLR) and treated with anti-VISTA-antibodies. **(A)** Impact of different isotype backbones (IgG1-WT, IgG1-KO, IgG4-Pro) on the effects of anti-VISTA-antibody clones (VSTB112, E008, J014) on TNF levels in MLR. Data is from one experiment. Symbol styles indicate different antibodies; each single connected symbol represents a donor pair. **(B)** Panel of cytokines and chemokines in MLR cultures treated with anti-VISTA-antibody J014 on three different isotype backbones (IgG1-WT, IgG1-KO, IgG4-Pro). Data is combined from multiple experiments with four unique donor pairs; shown is the median fold-change of cytokine levels between J014 on each backbone and the respective backbone isotype control. **(C)** Impact of blocking Fc-receptors on anti-VISTA-antibody effects (J014-IgG4-Pro in left panel, J014-IgG1-WT in right panel) in MLR. Data is from one separate experiment for each antibody, each connected symbol represents one donor pair. * indicates p < 0.05.

Next, we wanted to understand the impact of antibody design on function of these VISTA antibodies. The current state of the art choices for fully-human and humanized therapeutic antibodies are unaltered wild-type IgG1 (IgG1-WT), IgG4 with a stabilizing proline alteration (IgG4-Pro), and IgG1 with LALA alteration that widely abolishes Fc-receptor binding (IgG1-KO). All three isotype backbones were compared for the three VISTA antibody clones VSTB112, E008 and J014 (**Figure 3A**). VISTA antibodies on both IgG1-WT and IgG4-Pro led to increased levels of TNF in MLR cultures (compared to the respective isotype controls), while antibodies on IgG1-KO showed only markedly weaker effects, statistically significant for clones VSTB112 and J014. We further studied the cytokine profile induced by J014 on the three different backbones and observed that IgG1-WT and IgG4-Pro led to comparable though not identical cytokine release patterns (**Figure 3B**), matching the profile for IgG4-Pro already shown in **Figure 1B**. To clarify if that reduced activity of IgG1-KO indicates a requirement for Fc-receptor-interaction, we performed MLR cultures where the PBMCs were pre-incubated with Fc-receptor-blocking reagents. VISTA antibody J014 on both IgG4-Pro (**Figure 3C**, left panel) and IgG1-WT (**Figure 3C**, right panel) backbone depended on Fc-receptor binding. For both, CD64 was identified as the relevant Fc-receptor, as a specific antibody against CD64 reduced the effects of J014 down to base levels, similar as a peptide-based pan-Fc-receptor blocker (Fc-Block). Interestingly, anti-CD16-antibody seemed to have a general pro-inflammatory effect as it increased the base levels of TNF in the isotype control-treated MLR as well as in the J014-treated cultures. The used anti-CD16-antibody, clone 3G8 on mouse IgG1-kappa isotype (Biolegend), might display Fc-receptor interaction by itself, possibly causing the increased base levels of TNF. However, another anti-CD16-antibody, clone REA423 on recombinant human IgG1 isotype with negligible Fc-receptor binding (manufacturer’s information, Miltenyi Biotec), still demonstrated an increase of base TNF levels (data not shown), although not as strong as 3G8. Nonetheless, even in anti-CD16-antibody-treated cultures, J014 led to further increases of TNF. Blockade of CD32 by a pan-CD32-blocking antibody did not have an impact on the activity of either backbone. As CD32 has two forms with different functionality, CD32a acting mainly as a stimulatory receptor and CD32b as an inhibitory receptor, we assessed the effects of specifically blocking CD32a in a separate experiment. There, CD32a blockade similarly did not have an impact on the activity of anti-VISTA antibody J014-IgG4-Pro (data not shown).

**Figure 4:**
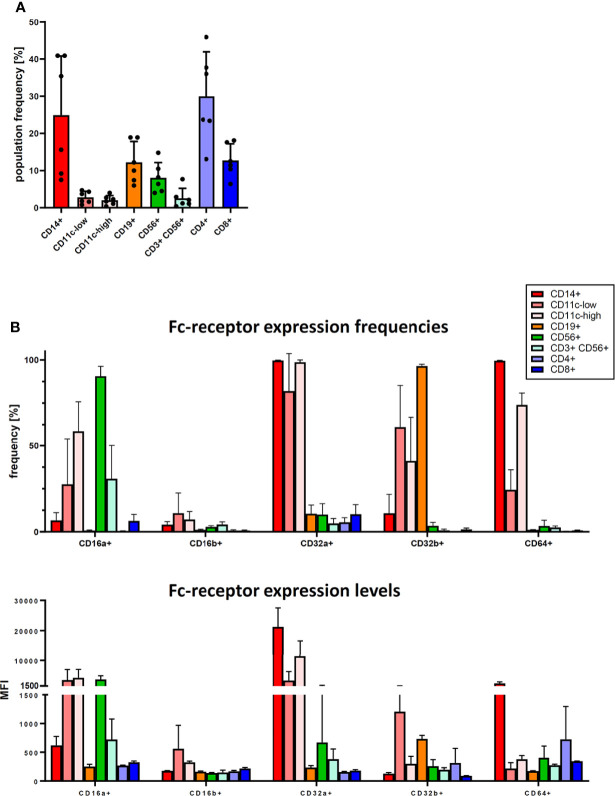
Fc-receptor profile of PBMC. Cryopreserved PBMC from six healthy donors were analysed for cell population frequency and Fc-receptor expression profile. **(A)** Frequency of major immune cell populations. Data are shown as bars of mean + SD, and as symbols indicating each separate donor. **(B)** Fc-receptor expression on the identified immune cell populations. Top panel: frequency of cells expressing a given Fc-receptor within the cell population, lower panel: median fluorescence intensity (MFI) of the Fc-receptor staining of these Fc-receptor-positive cells. Data are shown as bars of mean + SD. * indicates p < 0.05.

To further clarify the role of Fc-receptors, we studied their expression patterns on the major immune cell types found in human PBMC by flow cytometry. We identified several major immune cell populations in varying frequencies in the cryopreserved PBMC samples used in our study (**Figure 4A**): three myeloid cell populations: CD14+ cells (which are also CD11c-high), and CD11c-low and CD11-high cells (which are CD14-negative); a CD19+ B cell population; CD56+ CD3-negative NK cells; CD56+ CD3+ NKT cells; and CD4+ T cells and CD8+ T cell. These cell populations expressed Fc-receptors in different patterns. Most notably, CD64 was expressed nearly exclusively on the myeloid cell populations (**Figure 4B**, upper panel), with the highest expression level on CD14+ cells (**Figure 4B**, lower panel). These myeloid cell populations also expressed additional Fc-receptors – CD16a, CD32a and CD32b. While CD16a was also expressed on CD56+ NK cells, and CD32b on CD19+ B cells, CD16b was hardly expressed on any immune cell population.

**Figure 5:**
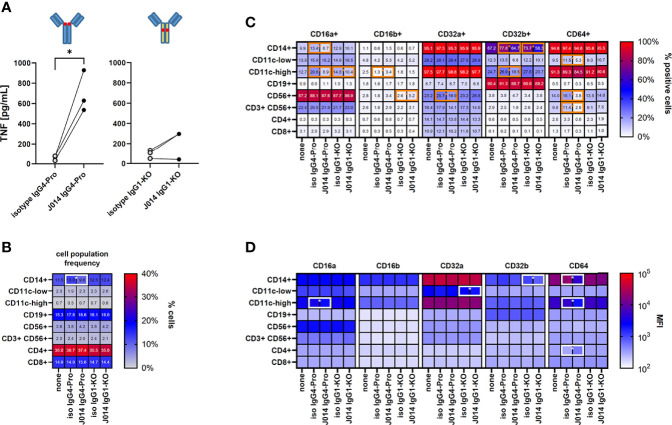
Fc-receptor profile of MLR cultures. PBMC from six healthy donors were co-cultured as three unique donor pairs to induce a mixed-leukocyte reaction (MLR), treated with anti-VISTA-antibody J014, and analysed for cell population frequency and Fc-receptor expression profile at the end of MLR culture. **(A)** Impact of anti-VISTA-antibody J014 on IgG4-Pro or IgG1-KO backbone on TNF levels in the MLR. Data is from one experiment; each single connected symbol represents a donor pair. **(B)** shows the frequency of major immune cell populations, **(C)** the frequency of Fc-receptor-positive cells within the identified immune cell populations, and **(D)** the Fc-receptor expression levels as median fluorescence intensity (MFI) of Fc-receptor-positive cells in MLR cultures either untreated (“none”) or treated with J014 on IgG4-Pro or IgG1-WT. Data are shown as mean values of three different donor pairs. Statistical significant differences between J014 antibody and its respective backbone isotype are indicated by a surrounding box. * indicates p < 0.05.

We then studied the impact of VISTA antibodies on Fc-receptor expression in the MLR system. We compared the VISTA antibody J014 on IgG4-Pro backbone to J014 on IgG1-KO backbone, and corresponding isotype controls. Thus, we were able to compare antibodies binding to VISTA and to Fc-receptors (via their Fc-backbone) in different strengths: (1) the isotype antibodies not binding to VISTA, and able to bind to Fc-receptors (as IgG4-Pro) or not (as IgG1-KO), and (2) J014 binding to VISTA and simultaneously to the Fc-receptors (as IgG4-Pro) or not (as IgG1-KO). **Figure 5A** confirmed that the antibodies performed in this experiment as expected and already shown in **Figure 3A**, with J014-IgG4-Pro leading to significantly enhanced levels of TNF. In those J014-IgG4-Pro-treated cultures, the frequency of CD14 cells was significantly reduced (**Figure 5B**). The most consistent changes were seen with the expression of CD64: The frequencies of CD64+ cells were significantly reduced within the CD11c-low population and the CD56+ NK cell population (**Figure 5C**), and the expression levels of CD64 were significantly reduced in the three myeloid cell populations (**Figure 5D**). We also observed some changes in the expression patterns of the other Fc-receptors, though not as consistent as for CD64. Unexpectedly, the presence of isotype antibodies led to an increase of CD32b+ cells within the CD14+ cell population as compared to the respective VISTA-binding antibodies.

### VISTA antibody interacts with CD16 to activate resting myeloid cells

**Figure 6:**
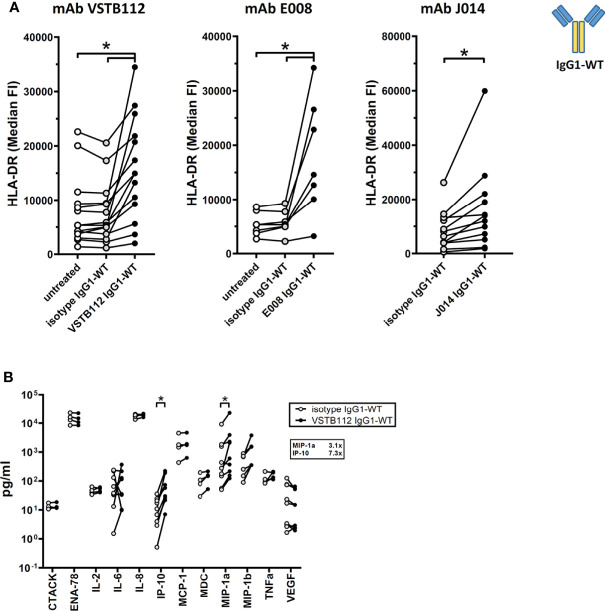
Effect of anti-VISTA antibodies on resting immune cells. PBMCs of single donors were cultured without stimulation and treated with anti-VISTA-antibodies. **(A)** Impact of anti-VISTA-antibody clones (VSTB112, E008, J014) with IgG1-WT isotype backbone on HLA-DR expression levels on monocytes. Data is combined from multiple experiments. Each connected symbol is a separate donor, some of which were used for testing multiple anti-VISTA antibodies. **(B)** Panel of cytokines and chemokines in cultures treated with anti-VISTA-antibody VSTB112-IgG1-WT. Data is combined from multiple experiments, each connected symbol represents a separate donor. * indicates p < 0.05.

**Figure 7:**
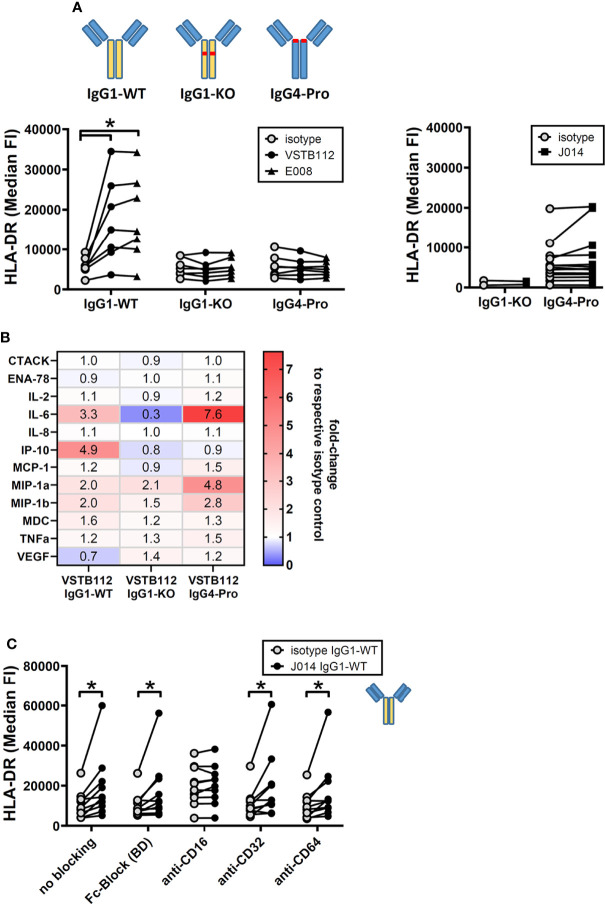
anti-VISTA antibodies effects on resting immune cells require Fc-receptor interaction. PBMCs of single donors were cultured without stimulation and treated with anti-VISTA-antibodies. **(A)** Impact of different isotype backbones (IgG1-WT, IgG1-KO, IgG4-Pro) on the effects of anti-VISTA-antibody clones (VSTB112, E008, J014) on HLA-DR expression levels on monocytes. Data is from multiple experiments, each connected symbol represents a separate donor; the data for VISTA antibodies with IgG1-WT backbone is also shown in (Figure 4A). **(B)** Panel of cytokines and chemokines in cultures treated with anti-VISTA-antibody VSTB112 on three different isotype backbones (IgG1-WT, IgG1-KO, IgG4-Pro). Data is from one experiment with four unique donors; shown is the median fold-change of cytokine levels between VSTB112 on each backbone and the respective backbone isotype control. The data of IgG1-WT has also been included in **Figure 6B**. **(C)** Impact of blocking Fc-receptors on the effects of anti-VISTA-antibody J014-IgG1-WT on HLA-DR expression levels on monocytes. Data is combined from multiple experiments, each connected symbol represents a separate donor; the data for J014 with IgG1-WT backbone without blocking is also shown in (**Figure 4A**). * indicates p < 0.05.

Another major interest when developing immune modulatory antibodies is their effect on a resting (i.e. non-activated) immune system. To study the effect of VISTA antibodies on a resting system, total PBMCs from one donor were cultured for 24h with VISTA antibodies, but without any other stimulation. The addition of VISTA antibody clones VSTB112, E008 and J014 on IgG1-WT backbone led to an increase in the levels of the activation marker HLA-DR on CD11b+ myeloid cells (**Figure 6A**). Using VSTB112-IgG1-WT as representative antibody, we furthermore observed that the chemokines IP-10 and MIP-1-alpha were increased in the cell culture supernatants (**Figure 6B**). Like the effects in MLR, the effects on HLA-DR depended on a particular isotype backbone. As shown in **Figure 7A**, the increase of HLA-DR levels is only observed with VISTA antibodies on IgG1-WT backbone, while IgG1-KO and also IgG4-Pro did not lead to an activation of resting myeloid cells. This difference in IgG1-WT and IgG4-Pro is also reflected in the cytokine release pattern (**Figure 7B**), where VISTA antibody VSTB112 on IgG4-Pro did not lead to an increase in IP-10. Still, VSTB112-IgG4-Pro induced the release of inflammatory cytokines such as IL-6, even though it did not lead to activation of myeloid cells (as measured by activation marker HLA-DR). However, in difference to the effects in MLR, the activation of myeloid cells by VISTA antibodies couldn’t be blocked by a pan-Fc-receptor blocker (Fc-Block), nor Fc-receptor-specific antibodies against CD64 or CD32 (**Figure 7C**), and also not by CD32a-specific blockade (data not shown). As in MLR, the anti-CD16-antibody 3G8 led to a general increase in baseline activation, here in form of increased HLA-DR-levels, even in the isotype control-treated culture wells. In contrast to the MLR system, anti-CD16-antibody prevented further effects by J014-IgG1-WT, thus indicating that CD16 might be a key Fc-receptor in this system.

Interestingly, while the effects of VISTA antibody seemed to be limited to myeloid cells in this culture system, purified monocytes were not activated by VISTA antibody on IgG1-WT backbone. The addition of purified NK cells, but not B cells or T cells, restored the effects of VISTA antibody on HLA-DR levels of myeloid cells (**Supplementary Figure 1**).

### Fc-receptor interaction is required for VISTA antibody effect on AML samples

**Figure 8:**
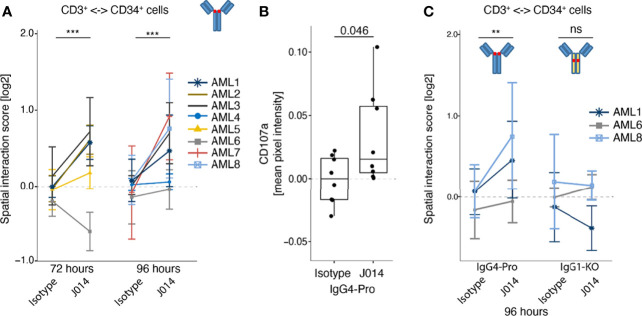
Effect of anti-VISTA antibodies in AML patient samples *ex vivo*. Bone marrow samples or PBMCs of AML patients were incubated *ex vivo* without stimulation, treated with various anti-VISTA-antibodies, and analysed by Pharmacoscopy. **(A)** Impact of anti-VISTA-antibody J014-IgG4-Pro on the interaction of CD3+ cells with CD34+ cells (left panel). Each symbol and color represents a distinct AML sample assayed in a separate experiment, described in the legend. Mean and SD shown for at least 3 technical replicates per sample and stimulation. **(B)** Mean pixel intensity of degranulation marker CD107a on CD3+ cell surface of a representative AML sample (AML #9 at 96h incubation time). Each symbol represents a technical replicate. **(C)** Impact of different isotype backbones (IgG4-Pro, IgG1-KO) on the effects of anti-VISTA-antibody J014 on the interaction of CD3+ cells with CD34+ cells. Each symbol and color indicates an AML sample assayed in a separate experiment, at 96h incubation time, see legend. Data of J014 on IgG4-Pro in **(C)** are also shown in (A, left panel). Mean and SD shown for at least 3 technical replicates per sample and simulation.** indicates p < 0.01, *** indicates p < 0.001, ns = "not significant".

To determine immune changes elicited by anti-VISTA antibodies within a cancer primed system, we used primary samples from patients with acute myeloid leukemia (AML) as a primary *ex vivo* model. Here, we assessed the J014-IgG4-Pro antibody as this showed the strongest functional effects in an ongoing immune response (MLR; **Figure 1A**) but did not demonstrate a general immune system activation (**Figure 7A**). One goal of immune-oncology drugs is to induce the interaction of activated effector cells to cancer cells, resulting in the cancer cell death. In this primary model, CD3+ cells within samples stimulated with J014-IgG4-Pro increased their interactions to CD34+ AML cancer cells (**Figure 8A**), and simultaneously an increase of surface CD107a expression was observed suggesting T cell activity (**Figure 8B**). As observed with MLR, the increased interaction score depended on the IgG4-Pro backbone: J014 on IgG1-KO backbone did not lead to increased interaction CD3+ T cells with CD34+ AML cancer samples (**Figure 8C**). The ability of VISTA antibody to not only activate an immune response but to also potentially drive effector cell functionally in a primary tumor sample model indicates potential therapeutic benefit of VISTA blockade.

## Discussion

In this study, we describe the functionality of three antagonistic antibody clones targeting the immune checkpoint molecule VISTA, and their dependency on particular isotype backbones. We selected antibodies that increased T cell-driven immune responses (using mixed leukocyte reactions as *in vitro* model system) and observed that they shared some properties: they bound to a similar region on the VISTA protein and showed a comparable functional profile. The isotype of the antibody backbone had a profound effect on antibody functionality: IgG1-WT allowed VISTA antibodies to increase ongoing immune reactions in the MLR as well as to activate resting myeloid cells in unstimulated cultures. IgG4-Pro VISTA antibodies, on the other hand, only affected the MLR system but did not activate resting myeloid cells. Using IgG1-KO as antibody backbone practically abolished all antibody functionality we observed in our assay systems. This lack of activity suggested that Fc-receptor-interaction is required for VISTA antibody functionality. That concept was confirmed for both systems, as Fc-receptor blockade prevented the effects of VISTA antibodies, namely CD64 in the MLR system, but CD16 in resting cultures. This difference in the involved Fc-receptors might explain why only VISTA antibodies on IgG1-WT backbone were able to activate resting immune cells. We further demonstrated that VISTA antibody on IgG4-Pro backbone increased the interaction of effector immune cells with CD34+ AML cancer cells in *ex vivo* patient samples; that effect was similarly reduced by an IgG1-KO backbone.

The pro-immunogenic effects of VISTA antibodies in the MLR fit to the notion of VISTA as immune regulatory molecule for ongoing immune reactions. However, it is unclear whether VISTA on myeloid cells or on T cells is more relevant in this system. We deliberately chose a system based on complete PBMCs with all major immune cell populations to reflect the complex human therapeutic setting *in vitro*, including interaction of multiple cell populations. Here, the effects of VISTA antibody depended not only on binding to VISTA, but also on Fc-receptor-binding. One possible reason could be the need to sequester VISTA away from the immunological synapse, as immune regulatory molecules are often integrated in the synapse close to the TCR and function by altering the TCR-signaling pathways (45). It has been shown that VISTA similarly reduces the phosphorylation of early TCR signaling events (46), suggesting that VISTA may also need to be in proximity of the TCR complex during TCR stimulation to achieve its immune modulatory effects. An antibody binding VISTA and simultaneously bound to Fc-receptor might prevent VISTA from entering the synapse and thus prevent immune modulation. In that respect it is not relevant if VISTA on T cells or VISTA on myeloid cells is sequestered, as both cells are part of the immunological synapse. The Fc-receptor expression patterns support the notion of myeloid cells being directly involved in the effects of VISTA antibodies in our MLR system. In PBMC, CD64 is expressed nearly exclusively on myeloid cells, especially high on CD14+ monocytes, and the expression levels are reduced upon treatment with VISTA antibodies on IgG4-Pro backbone; we did not investigate if this is based on reduced expression, increased shedding and/or increased internalization of CD64. This CD64 reduction was not observed with isotype IgG4-Pro or VISTA antibody on IgG1-KO backbone, suggesting that a simultaneous binding of VISTA and CD64 is required. Interestingly, Fc-receptor binding is also an important aspect for other immune modulatory antibodies (47), with antagonistic anti-CTLA-4 antibodies requiring Fc-receptor-binding during APC-T cell interaction for impacting TCR signaling (48).

The effects of antagonistic VISTA antibody on resting immune cells point towards a different mode of action. First, only IgG1-WT antibodies showed an effect on myeloid cell activation markers, but not IgG4-Pro antibodies, while both led to increased cytokine release (albeit different cytokine profiles). Second, the effect required a different class of Fc-receptor. Third, the effect seems to be limited to activating myeloid cells. The last aspect is maybe the easiest to discuss: myeloid cells are known to express VISTA at high levels and that expression is fairly stable, while T cells seem to lose VISTA rapidly *in vitro* (16). Hence, myeloid cells might be the main cells left to target. VISTA might act here as a regulator for keeping naïve non-activated immune cells silent, as suggested by ElTanbouly et al. (12, 26) The blockade of VISTA might then release a brake for myeloid cells, which enter an activated state. However, these effects required not only binding to VISTA, but also interaction of an IgG1-WT backbone with Fc-receptor CD16, and the presence of NK cells, which express CD16a. This could again be explained by the concept that VISTA needs to be clustered and/or sequestered away to prevent its immune inhibitory effects, probably in trans, as the CD16 expressed on myeloid cells does not enable this effect. This particular aspect of the dependency on IgG1-WT backbone and NK cells has been presented previously for the clinical trial candidates VISTA antibody CI-8993 (formerly JNJ-61610588) (49, 50) and KVA12.1 (51). Another study similarly demonstrated that myeloid cells were activated upon incubation with anti-VISTA antibodies on human IgG1-WT backbone, but not when these clones (KO11-1B1 and VIBE1A) were placed on an IgG1-variant with reduced Fc-effector function (mutations L234A, L235A, P324G) (9).

When we added VISTA antibody on IgG4-Pro backbone to *ex vivo* AML patient samples, we observed an increase in the interaction of T cells with CD34+ AML cancer cells. This interaction again depended on the backbone, similar to our MLR system. In that respect, the AML samples reacted like cultures with an ongoing immune reaction, not like unstimulated resting PBMCs. This is an intriguing similarity, as it points to the possibility that the endogenous anti-tumor immune reaction of cancer patients can be increased by VISTA-antibody. Interestingly, a recent study demonstrated an increase of TNF levels in human clear cell RCC tumor slice *ex vivo* cultures by incubation with the anti-VISTA antibody clone 311-H7 (on mouse IgG1 backbone) (34).

An important question is the capacity of VISTA antibodies to induce cytokine release syndrome similar to other immunotherapy strategies in oncology. This is difficult to gauge based on our *in vitro* systems, as they probably do not reflect the complex human *in vivo* situation. Nonetheless, it is notable that in our systems, resting immune cells were activated in a different pattern by VISTA antibodies on IgG4-Pro as compared to IgG1-WT backbone: myeloid cells did not increase their activation marker HLA-DR, and IP-10 levels were also not increased. However, levels of other pro-inflammatory cytokines like IL-6 did increase. In a recent study, the VISTA antibody HMBD-002 (binding to a different epitope on VISTA than the antibodies in our study) on IgG4-Pro was also assessed in a MLR system, and showed a pro-inflammatory cytokine pattern with some similarity to our results (increases in TNF alpha, IFN-gamma and IL-6), but also differences (increases in IL-4, IL-10, IL-13, IL-17 and IL-23). Of note, that antibody was subsequently assessed in human whole blood assays as well as human PBMC studies and did not show any significant effects on the levels of IL-2 and IL-6 (33).

Our study focused on elucidating the impact of antagonistic anti-human VISTA antibodies on human primary cells, to increase our understanding for designing possible therapeutic compounds. Other published studies additionally employed mouse model systems of syngeneic tumors to assess the anti-tumor effects of VISTA antibodies. Multiple different clones on different isotype backbones were used, most notably clone 13F3 on hamster IgG (16, 29) and also clones MH5A (hamster IgG) (27), MIH63 (rat IgG2a) (28, 31), VISTA.10, VISTA.16 and VISTA.18 (mouse IgG1-D265A) (2), and SG7 (mouse IgG2a and IgG2a LALA/PG) (6). Those mouse studies showed in general some weak effects by the VISTA antibody, which could be increased by combination with other therapeutical interventions. A recent study demonstrated tumor growth inhibition in syngeneic as well as in humanized mouse tumor models by the anti-VISTA antibody clone HMBD-002, described to cross-react with human, non-human primates, rat and mouse VISTA, on human IgG4-Pro backbone, and clone V4P, a precursor form of HMBD-002, on both mouse IgG2a and IgG2a LALA/PG backbones (33). The impact of the isotype backbones in those studies is difficult to gauge, as the clones probably have different binding characteristics to VISTA, have vastly different backbones (even from non-mouse species) and were not tested side by side in one experiment. Furthermore, function, expression and binding of Fc-receptors are different in mouse compared to human (52). Hence, it is unclear if mouse *in vivo* systems are a good model to study isotype backbone effects of human candidates for therapeutic antibodies. Nonetheless, VISTA.10, VISTA.16 and VISTA.18 (all on IgG1-D265A with reduced Fc-receptor binding) showed weak anti-tumor effects (2), and SG7 as well as V4P on both a wild-type mouse IgG2a and a LALA/PG variant with reduced Fc-receptor-binding demonstrated comparable anti-tumor effects (6, 33), suggesting that Fc-receptor interaction may not play a major role in these mouse tumor models.

An open question is the functional binding partner of VISTA. Multiple molecules are under discussion, such as PSGL-1, VSIG-3, Galectin-9 and Syndecan-2, and these might all play a role under different circumstances, such as different pH in the microenvironment (2, 4, 8, 9). A recent publication studied the binding sites of VSTB112 and other antagonistic VISTA antibodies (6). All three tested antibodies (VSTB112, BMS767 and SG7) cross-competed with each other when binding VISTA, and all three antibodies were capable of blocking PSGL-1 and VSIG3 from binding to VISTA. While the major binding amino acids were not identical for those antibodies (6), all of them fell into or adjacent to the area that we identified for VSTB112, as well as for our clones J014 and E008, in our HDX protection assay (**Table 1**). Yet, as the isotype backbone of the antibody strongly impacted the functional activity of VISTA antibodies in our study, ligand blockade does not seem to be the only relevant aspect of their mode of action. This is also supported by findings that clones KO11-1B1 and VIBE1A bind to different non-competing epitopes on VISTA, but show similar functional effects and dependency on Fc-effector function. However, no further information on the involved epitope, binding partner or Fc-receptor was provided in that study (9). HMBD-002 is another anti-VISTA antibody binding to a different epitope. It is described to block VSIG-3, but not PSGL-1 ligand binding. HMBD-002 was shown to bind to the residues 69-97 on VISTA, which differs from the binding region of the clones used in our study. The authors chose an IgG4-Pro backbone but did not assess possible functional interaction of that format with Fc-receptors (33). Given these similarities in binding and functional effects of multiple published VISTA antibodies, the particular interactions of antibody backbones and Fc-receptors observed in our study may well be universally applicable to all immune stimulatory VISTA antibodies. However, with multiple possible VISTA binding partners, multiple Fc-receptors, and impact of pH on the interaction of VISTA with its binding partners as well as antibody with Fc-receptors, the overall effect in humans is difficult to assess.

In conclusion, we demonstrate here that the isotype backbone can have a profound impact on the functional profile of antagonistic VISTA antibodies. Hence, it is important to study possible candidate therapeutic antibodies on different isotype backbones to understand their mode of action and to select the best antibody design for clinical efficacy accordingly.

## Data availability statement

The raw data supporting the conclusions of this article will be made available by the authors, without undue reservation.

## Ethics statement

The studies involving human patients were reviewed and approved by Ethics Commission of the Medical University of Vienna. The patients/participants provided their written informed consent to participate in this study.

## Author contributions

SM, HW, TF, RK-B, AV designed experiments. SM, HW, TF, BR, SS, YH, GH acquired and analysed data. SM, HW, TF, RK-B, IT, AV wrote the paper. SM, HW, TF, RK-B, AV supervised research. SM, AV conceived of the project. SM is the guarantor of this work. All authors contributed to the article and approved the submitted version.

## Funding

This study received funding from the Österreichische Forschungsförderungsgesellschaft (grant# 852068). In addition, Boehringer Ingelheim provided funding for this study,

## Acknowledgements

We thank Daniela Fürweger, Claudia Reichel-Voda, Ilse Apfler, Irene Schweiger and Thomas Prenninger for technical assistance, and Gregory Vladimer and Christina Taubert from Allcyte GmbH in Vienna, Austria for their work using Pharmacoscopy on AML patient samples.

## Conflict of interest

All authors were employees of Boehringer Ingelheim at time of this study. All authors declare no other competing interests.

## Publisher’s note

All claims expressed in this article are solely those of the authors and do not necessarily represent those of their affiliated organizations, or those of the publisher, the editors and the reviewers. Any product that may be evaluated in this article, or claim that may be made by its manufacturer, is not guaranteed or endorsed by the publisher.

## Author disclaimer

The funder was not involved in the study design, collection, analysis, interpretation of data, the writing of this article or the decision to submit it for publication.
