# Supplementary material for: Distinct immune stimulatory effects of anti-human VISTA antibodies are determined by Fc-receptor interaction
Source: Front Immunol. 2022 Jul 28;13:862757. doi: 10.3389/fimmu.2022.862757 (PMC9367637; doi:10.3389/fimmu.2022.862757)
Supplement: Supplementary file 1 [file DataSheet_1.pdf]

Supplementary Material

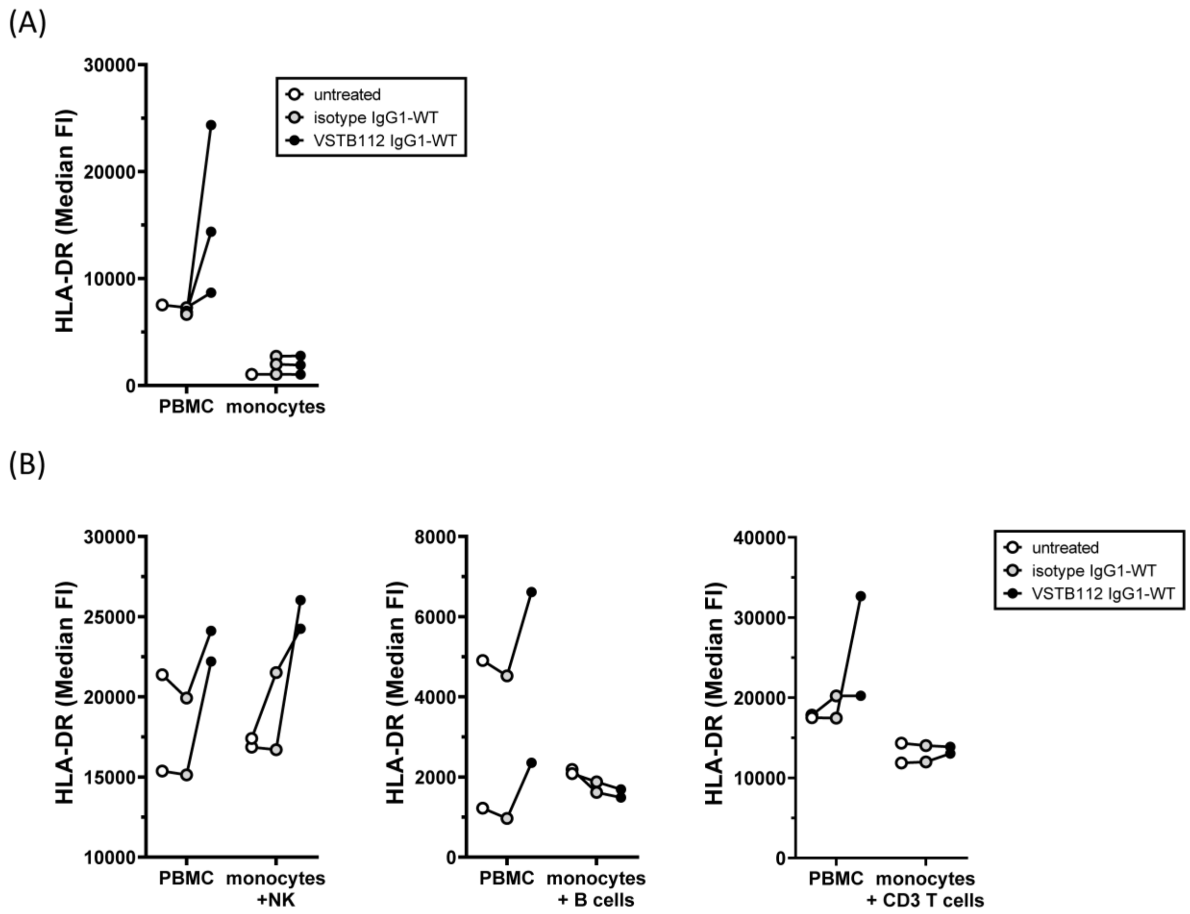

Supplementary Figure 1. Cell populations relevant for in vitro functionality of VISTA antibodies

(A) Total PBMC or enriched monocytes were treated with anti-VISTA-antibodies VSTB112 with IgG1-WT backbone. Data are from multiple experiments; each connected symbol indicates a separate donor.

(B) Indicated cell populations were separated from total PBMC, re-combined (within the same donor) and cultures treated with anti-VISTA-antibodies VSTB112 with IgG1-WT backbone. Data are from multiple experiments using the same two donors; each connected symbol indicates a separate donor.
